# New Dihydroxytyrosyl Esters from Dicarboxylic Acids: Synthesis and Evaluation of the Antioxidant Activity In Vitro (ABTS) and in Cell-Cultures (DCF Assay)

**DOI:** 10.3390/molecules25143135

**Published:** 2020-07-09

**Authors:** Elia Roma, Elena Mattoni, Paolo Lupattelli, Seyed Sepehr Moeini, Tecla Gasperi, Roberta Bernini, Sandra Incerpi, Daniela Tofani

**Affiliations:** 1 Department of Science, Roma Tre University, Viale G. Marconi 446, 00146 Rome, Italy; elia.roma@uniroma3.it (E.R.); seyedsepehr.moeini@uniroma3.it (S.S.M.); tecla.gasperi@uniroma3.it (T.G.); sandra.incerpi@uniroma3.it (S.I.); 2 Centro Interdipartimentale per la Didattica Chimica (CeDiC), Via della Vasca Navale 79, 00146 Roma, Italy; elena.mattoni@uniroma3.it; 3 Department of Science, University of Basilicata, Via dell’Ateneo Lucano 10, 85100 Potenza, Italy; paolo.lupattelli@unibas.it; 4 Department of Agriculture and Forest Sciences (DAFNE), University of Tuscia, Via S. Camillo de Lellis, 01100 Viterbo, Italy; roberta.bernini@unitus.it

**Keywords:** antioxidants, hydroxytyrosol, hydroxytyrosyl esters, bioactive constituents, nutraceuticals, ABTS assay, DCF assay, structure–activity relationship

## Abstract

New dihydroxytyrosyl esters **2a, 2c–2j** of dicarboxylic acids were synthesized from methyl orthoformate protected hydroxytyrosol **3** and diacyl chlorides. New compounds were characterized (HRMS, FT-IR, ^1^H- and ^13^C-NMR), and tested for antioxidant activity both in vitro (ABTS) and on L6 myoblasts and THP1 leukemic monocytes cell culture by DCF assay. According to the ABTS assay, compounds **2a, 2c–2j** showed a TEAC value of antioxidant capacity up to twice that of Trolox. Very high or complete ROS protections were obtained in the cell environment where lipophilicity and rigidity of dicarboxylic structure seem to facilitate the antioxidant effect. MTT assay and proliferation test were used for assessment of cell viability. These compounds can be envisaged as a new class of preservatives for food or cosmetic products.

## 1. Introduction

Hydroxytyrosol **1** (HTyr), a natural polyphenolic antioxidant, was recently recommended by EFSA as a food supplement in a balanced diet for its important health benefits [1], and is the ideal candidate to answer to the compelling demands for new nutraceuticals and safe natural preservatives [2]. A recent study highlighted the potential therapeutic effects of HTyr [3] due to its bioavailability [4] and biological properties ranging from antiaging protection of cells [5,6], antimicrobial activity [7], neuroprotective effects [8] and protection against cardiovascular disease [9], cancer [10,11], and HIV [12]. Unfortunately, its human health benefits collide with its high reactivity and low solubility in a lipophilic environment. To overcome these drawbacks, lipophilic HTyr derivatives were synthesized and extensively studied in recent years [13,14]. These compounds demonstrated an in vitro antioxidant activity comparable to the natural precursor, with higher fat solubility that could enhance their bioavailability and pharmacodynamic profile. 

On the other hand, as many phenolipids, in cells, they drew the attention to the dependence of antioxidant activity as a function of the fatty acid chain length. This produces a parabolic trend, the so-called *cut off effect* [15,16], with drastic activity decrease with long alkyl chain Htyr esters. As a matter of fact, long chain fatty acid esters of HTyr show high affinity to membrane, so preventing the oxidation directly inside the bilayer, but hardly enter the cytosol and seem not useful as preservatives due to low solubility in water and tendency to produce micelles [17,18]. 

Utilizing HTyr esters of dicarboxylic acid could be a new strategy to partially solve this dichotomy through the enhancement of lipophilicity abating the micellization problem. The dicarboxylic acid structure could modulate not only the lipophilicity of the final compound, but also the distance between the hydroxytyrosyl moiety and the rigidity of the structure. These last features might be helpful to understand if a correlation between the acidic structure and antioxidant activity could be drawn. Furthermore, in these esters, using equimolar quantity of substrate, the antioxidant capacity should be increased, since two catecholic functions are present. 

In this paper, the synthesis of new dihydroxytyrosyl dicarboxylic acid esters and the determination of their antioxidant activity both in vitro and in cell cultures are reported, the cell viability is analyzed and the structural variability of the acidic moiety is utilized to rationalize some structure–activity correlations.

## 2. Results and Discussion

### 2.1. Synthesis of Dihydroxytyrosyl Esters

To the best of our knowledge, HTyr esters derived by dicarboxylic acid have never been synthetized, nor studied as antioxidants. Aiming at this goal and to better understand the eventual effect of the dicarboxylic acid structure on the antioxidant activity, ten dicarboxylic acids were chosen: from oxalic to glutaric (**2a–e**, C2–C6) and suberic (**2f**, C8) to analyze the effect of aliphatic chain elongation, while trans-1,4-cyclohexandicarboxylic (**2g**), fumaric (**2h**), phthalic (**2i**), and isophthalic acids (**2j**) to take into account the effect of an enhanced rigidity of the structure (Table 1).

The synthesis of hydroxytyrosyl esters generally suffers a few drawbacks: (i) the presence of alkylic and phenolic alcohols can compete to the esterification reaction with acyl chlorides; and (ii) the catecholic group oxidation during the work-up and purification can decrease the yields. To avoid these problems, the esterification could be performed using both enzymatic or chemical procedures [19,20]. As an example, HTyr nicotinate and HTyr lipoate were synthetized by chemical procedures including the protection and then the deprotection of the catecholic moiety of HTyr [21,22], while lipophilic HTyr esters were generally prepared enzymatically using *Candida antarctica* lipase B [23,24]. As an alternative, recently in our laboratories, two chemical procedures have been optimized to obtain lipophilic HTyr esters. The first is based on the direct esterification of tyrosol with acyl chlorides of different chain length, followed by oxidation of the phenolic rings to achieve the catechol moieties [25,26]; the second on the preliminary protection of the catecholic moiety of HTyr as methyl-orthoformate, followed by esterification reaction with acyl chlorides and then deprotection [13,27].

**Scheme 1:**
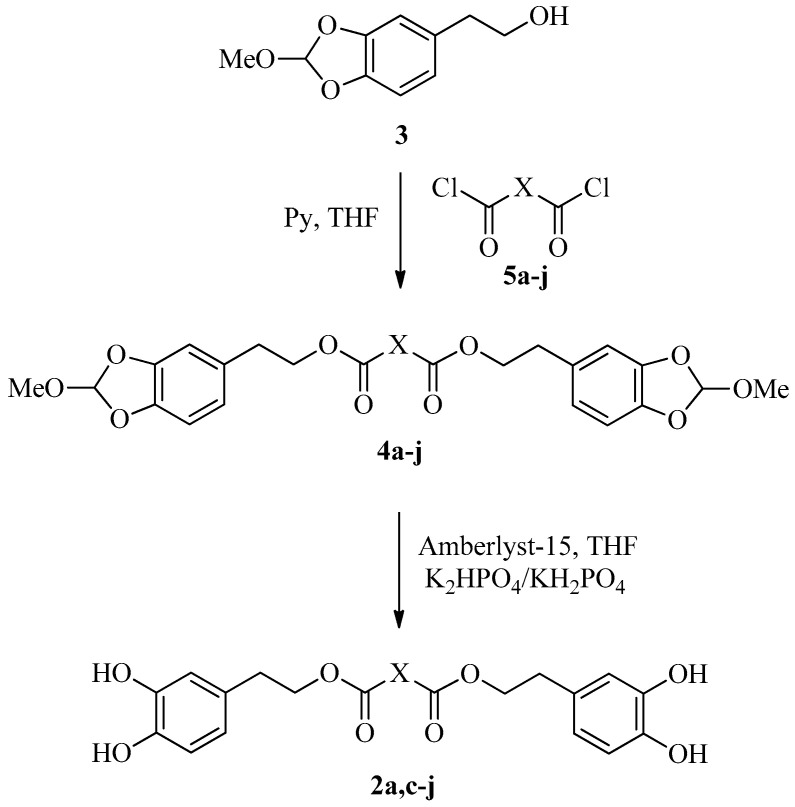
Synthetic route to obtain dihydroxytyrosyl esters **2a–2j**.

Based on our experience, the first pathway, even if very simple and direct, is not applicable to the synthesis of dihydroxytyrosyl derivatives, for the presence of two catecholic groups in the final products, which could enhance the oxidative degradation during the purification process and lower the yields of reaction. On the contrary, the second way, even if it takes longer, could lead to obtainment and purification of stable protected dihydroxytyrosyl esters, and to deprotect them under mild conditions without the requirement of further purification. On the basis of these considerations, methyl orthoformate protected HTyr **3** was easily synthetized as reported in the literature [27]. The protected dihydroxytyrosyl dicarboxylic esters derivatives **4a–4j** were obtained by a reaction of **3** with the corresponding diacyl chlorides **5a–5j** in anhydrous THF in the presence of two equivalents of pyridine (Scheme 1). 

The main drawback of this synthetic pathway is the need for high diacyl chloride purity. The presence of free dicarboxylic acid, derived by partial hydrolysis of diacyl chloride, could produce the corresponding free HTyr **1** by deprotection of **3** during the reaction and, even if it leads directly to the corresponding dihydroxytyrosyl ester, the yield reduces due to its partial oxidation during the purification step. Taking this into account, the acyl chloride was distilled just before use. The protected derivatives **4a–4j** were obtained, after chromatographic purification over pre-washed silica gel, in good yields for all the esters **4a, 4c–4j** (75–81%) with the exception of the moderate conversion to **4b** (55%). 

The deprotection of **4a–4j**, following the previously optimized procedure using Amberlist 15 acidic resin and phosphate buffer [13], gave the free dihydroxytyrosyl esters **2a, 2c–2j** in high yield (90–95%, Scheme 1) with the exception of malonate derivative **4b** that, in the reaction conditions, was rapidly degraded producing a black pitch. Either **4a–4j** and **2a, 2c–2j** were totally characterized (HRMS, IR, ^1^H- and ^13^C-NMR). 

### 2.2. ABTS Spectrophotometric Assay

**Figure 1:**
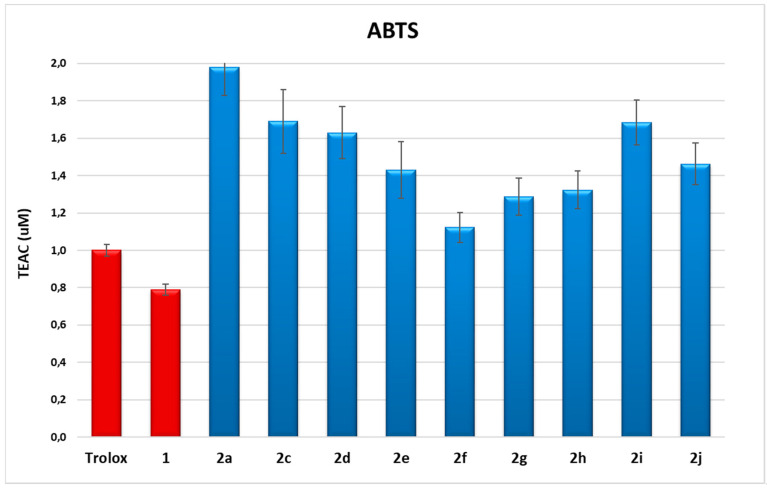
ABTS assay of HTyr **1** and dihydroxytyrosyl esters **2a, 2c–2j**. Data are presented as TEAC values (μM). Student’s *t*-test was used to calculate errors. One-way ANOVA and Bonferroni post-test were performed as statistical analysis (*p* < 0.05).

Compounds **1** and **2a, 2c–2j** were first analyzed for their antioxidant capacity in vitro by 2,2′-azino-bis(3-ethylbenzothiazoline-6-sulfonic acid) (ABTS spectrophotometric assay) [13,28]. The antioxidant capacities were measured in ethanol within a concentration range of 1–10 μM. The linear regressions of dose−response curves of each compound were calculated, and Trolox equivalent antioxidant capacities (TEAC) were obtained by comparison with data of Trolox, chosen as reference antioxidant. The data are reported in Figure 1.

These data indicate the high antioxidant capacity of all compounds tested, in many cases up to twice the antioxidant capacity of Trolox and of HTyr **1**. The major results were obtained from compounds with short chains between acyl groups, such as saturated dihydroxytyrosyl oxalate **2a** and succinate **2c**, having TEAC value of 2.0 ± 0.2 and 1.7 ± 0.2, respectively, or unsaturated dihydroxytyrosyl phthalate **2i** or isophthalate **2j**, with TEAC value of 1.7 ± 0.1 and 1.5 ± 0.1, respectively. To better understand a potential relationship between antioxidant capacity and lipophilicity, Log P data were calculated (Table 2). 

While poor linearity was obtained considering all products, TEAC values of alkylic diesters **2a, 2c–2g** proved to be inversely dependent to Log P, showing good linear correlation (Supplementary Material, Figure SM1, R^2^ = 0.858). Data evidence that longer alkylic chain between carboxylic moiety can be considered detrimental to antioxidant activity. Notably, the antioxidant capacity of compounds with the same number of carbons between the acyl groups present similar antioxidant activity, regardless of the rigidity of dicarboxylic structure. This behavior could be explained supposing that, in the diesters with longer carbon chains between the two acyl groups, conformational equilibrium could favor polar aromatic rings to get closer (π–π staking), thus partially forbidding both catechol groups to perform the antioxidant effect. 

### 2.3. ROS Determination in Cell Culture

**Figure 2:**
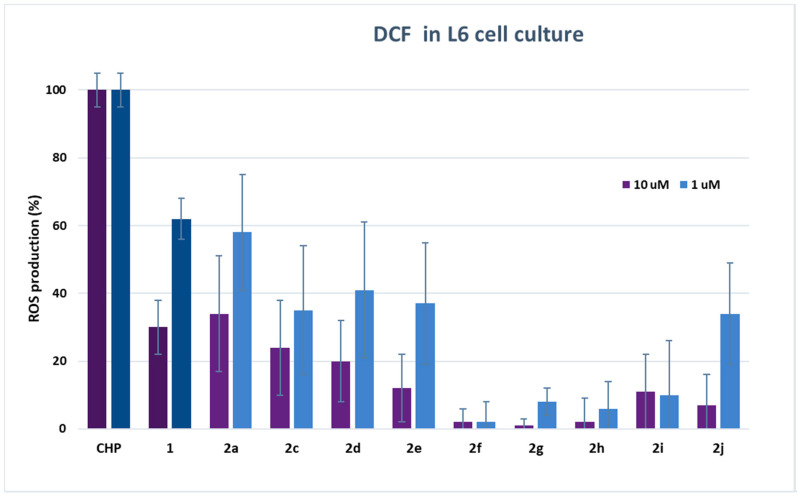
DCF fluorescence assay. Measure of ROS production in L6 myoblasts after stimulation with 200 µM CH with or without the presence of HTyr **1** or dihydroxytyrosyl esters **2a, 2c–2j** (10 and 1 µM). Each piece of data is the mean value of five experiments performed in triplicate ± SD. One-way ANOVA and Bonferroni post-test were carried out as statistical analysis.

**Figure 3:**
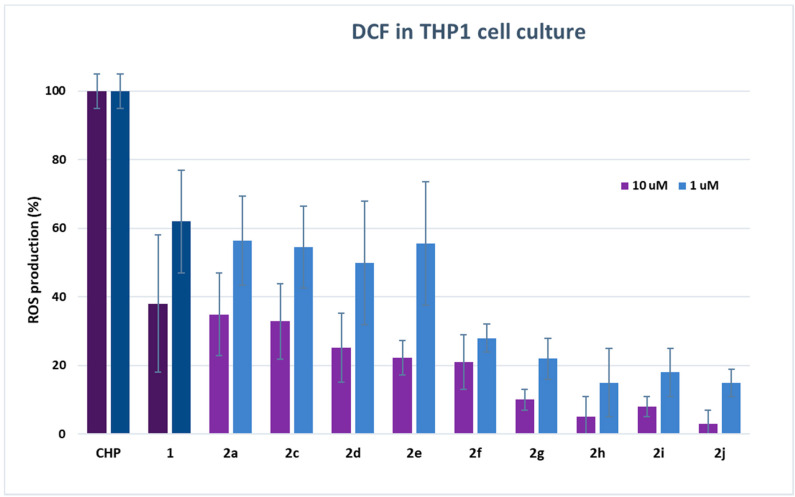
DCF fluorescence assay. The measure of ROS production in THP-1 monocytes after stimulation with 200 µM CH with or without the presence of HTyr **1** or dihydroxytyrosyl esters **2a, 2c–2j** (10 and 1 µM). Each piece of data is the mean values of five experiments performed in triplicate ± SD. One-way ANOVA and Bonferroni post-test were carried out as statistical analysis.

To determine the radical scavenging capacity of dihydroxytyrosyl esters in the cell environment, dichlorofluorescein radical (DCF) assay was carried out [30]. Fluorescent probe DCF radical was produced by oxidation of dichlorofluorescein (DCFH_2_) via cumene hydroperoxide (CH). The analyses on cells were performed both on L6 cells derived from rat skeletal muscle, particularly sensitive to oxidative stress, and on THP-1 monocytes, a human cell line of leukemic origin. The results of DCF assay are shown in Table SM1 (see Supplementary Material), and in Figure 2 and Figure 3, respectively, for L6 and THP-1 cell lines at 10 and 1 μM concentration of antioxidants.

Results either on L6 or THP1 cell cultures have provided evidence of a very high antioxidant activity of all tested compounds even with respect to HTyr **1**. In L6 cells, there was a decrease of ROS production up to 99% in comparison with cells treated only with CH. In particular, the best performances were obtained from dihydroxytyrosyl esters **2g**, **2f** and **2h** with, respectively, 1 ± 2%, 2 ± 4% and 2 ± 7% ROS production at 10 μM, and 8 ± 4%, 2 ± 6% and 6 ± 8% ROS production at 1 μM antioxidant concentration. The antioxidant activity of dihydroxytyrosyl esters in THP1 cell cultures showed a lower but still high ROS protection even with respect to HTyr **1**. In this case, compounds **2j** and **2h** behaved better with 3 ± 4% and 5 ± 6% ROS production at 10 μM, while, at 1 μM concentration, the best results were 15 ± 4% for both antioxidants (Figure 3).

In cell culture, lipophilicity seems to favor the antioxidant activity: data of DCF in L6 cells at 10 μM evidence a direct linear correlation between lipophilicity and antioxidant activity in linear esters **2a, 2c–2f** (R^2^ = 0.852, data not presented). However, the higher antioxidant activity of cyclic and aromatic compound is more difficult to rationalize by SAR. The different behavior of hydroxytyrosyl antioxidants **2g–2j** in vitro and on cell culture can let us suppose that, in the biological environment, other effects play a determining role in the antioxidant activity. As previously demonstrated for hydroxytyrosyl esters derived by linear carboxylic acids, the differences of antioxidant activity on cell cultures can be ascribed to a combination of factors: the higher lipophilicity favors the antioxidant to be transported in the cell membrane by passive diffusion but can block the phenolipid inside the bilayer [17,18], so better performances are obtained by medium chain esters [13]. In case of dihydroxytyrosyl esters **2g–2j**, the higher lipophilicity could enable penetration into cell membrane, while a more rigid antioxidant structure could facilitate the passage into the cytosol. Once inside the cell, the antioxidant activity seems to be related to both catechol groups, explaining the very high ROS protection obtained from these products.

### 2.4. MTT Assay and Cell Proliferation

**Figure 4:**
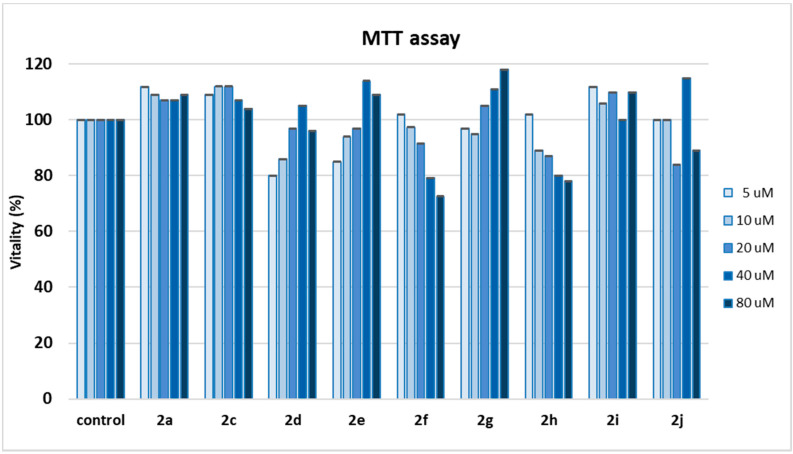
MTT spectrophotometric assay of compounds **2a, 2c–2j** at 10 to 80 µM concentration in L6 cells. Data of each compound represent the mean values of five analyses ± SD. One-way ANOVA and Bonferroni post-test were used as statistical analysis. *p* < 0.05 was considered a sufficient difference between values.

The high antioxidant activity observed for all dihydroxytyrosyl esters **2a, 2c–2j** prompted us to examine their cytotoxicity, in order to evaluate their potential utilization as preservatives in food or cosmetics application. 3-(4,5-dimethylthiazol-2-yl)-2,5-diphenyltetrazolium bromide (MTT) colorimetric assay was used as viability test in cell culture. L6 myoblasts were treated with antioxidants, in a concentration range from 5 to 80 µM, for 48 h (Figure 4). 

Cells that remain viable after exposure and incubation with **2a, 2c–2j** are able to reduce yellow water-soluble MTT salt into insoluble blue metabolite formazan that can be quantified spectrophotometrically and is directly related to the number of viable cells. The results have revealed a percentage of cell survival generally above 90% for all compounds analyzed. Interestingly, in the case of **2d**, **2e**, and **2g**, a hormetic effect was discovered, with better cell vitality at higher concentration of antioxidant, while with **2a**, **2c**, and **2i**, cell vitality resulted even enhanced at all concentrations of antioxidant, in comparison with control cells exposed only to solvent. 

**Figure 5:**
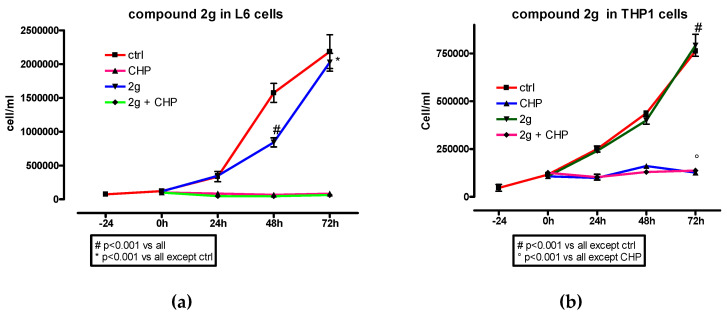
Effect of dihydroxytyrosyl ester **2g** (10 µM) on L6 (**a**) and THP-1(**b**) cell proliferation with or without CH (40 µM in L6 and 200 µM in THP-1 cells). Each compound was tested twice. Data are presented as mean values ± SD. One-way ANOVA and Bonferroni post-test was used as statistical analysis.

Usually, esters can be harmful by itself or due to their hydrolysis products obtained by reaction with cell enzymes. Hydrolysis of **2a, 2c–2j** leads to dicarboxylic acid and HTyr that has been demonstrated not only to be harmless but even protective. **2a, 2c–2e** are derived from linear dicarboxylic acids that are metabolic intermediate, while **2f–2j** contain dicarboxylic acids that are not natural and sometimes even toxic, like phthalic and isophthalic acids present in **2i–2j**. This choice derived, as already specified, by a precise interest in structure–activity relationship. However, the high antioxidant activity of dihydroxytyrosyl derivatives **2f–2j** encouraged us to further analyze the toxicity of the compounds synthesized. To perform cell proliferation experiments, both L6 myoblasts and THP-1 monocyte were used. The effect of compounds **2f–2j** was analyzed either in the presence or in the absence of CH. The cell proliferations in the presence of the dihydroxytyrosyl ester **2g** are reported here (Figure 5) while data of **2f, 2h–2j** are presented as Supplementary Materials (Figures SM2–SM5). 

In L6 cells, dihydroxytyrosyl esters **2h** and **2g** had no effect in cell proliferation while a smooth decrease was detected when **2f**, **2i** and **2j** were used. In case of THP-1 cell lines, **2f**, **2g** and **2j** did not influenced the cell proliferation, while **2i** showed a reduction and **2h** even an enhancement of cell growth with respect to control. In all cases, in the presence of CH, the antioxidants did not have a visible effect on the cell proliferation profile.

## 3. Materials and Methods

### 3.1. Reagents

All chemicals used were of analytical grade. Solvents and reagents were purchased from Sigma Aldrich (now Merck, Dusseldorf, Germany). Acyl chlorides **5a–5j** were distilled before use. HTyr **1** [31] and 2-(2-methoxy-benzo[1,3]dioxol-5-yl)-ethanol **3** were synthesized as described in the literature [27]. Silica gel 60 F254 plates and silica gel 60 were purchased from Merck (Milan, Italy). 2′,7′-Dichlorodihydrofluorescein diacetate (DCFH_2_-DA) was obtained from Molecular Probes (Eugene, OR, USA). L6 rat skeletal myoblasts and THP-1 monocytes were from the American Type Culture Collection (Rockville, MD, USA). Dulbecco’s modified Eagle’s medium (DMEM), antibiotics, and sterile plasticware were from Flow Laboratory (Irvine, CA, USA). Fetal bovine serum was from GIBCO (Grand Island, NY, USA). 

### 3.2. Instruments

^1^H- and ^13^C-NMR spectra were recorded in deuterated solvents (CDCl_3_, CD_3_OD 99.8% in deuterium) using a 400 MHz NMR spectrometer (Bruker, Munich, Germany). All chemical shifts were expressed in ppm (δ scale) and referenced to either the residual protons or carbon of the solvent. FT-IR spectra were recorded in CHCl_3_ on a Bruker Vector 22 spectrometer. HRMS were recorded with a Micromass Q-TOF mass spectrometer (Waters Corporation, Milford, MA, USA). VICTOR 3V Perkin Elmer spectrofluorometer was used to carry out fluorometric analyses. 

### 3.3. Synthesis of Protected Dihydroxytyrosyl Esters ***4a–4j***

In addition, 200 mg (1.020 mmoles) of **3** were dissolved in 4 mL of anhydrous THF, and 1.6 equivalents of pyridine and 0.8 equivalents of the freshly distilled acyl chloride (**5a–5j**) were added. The solution was left under argon atmosphere in the dark at room temperature, monitoring the progress of the reaction by HPLC analyses. After 4 h, the reaction solution was diluted with 2 mL of *brine* to destroy acyl chloride excess, and the eventual acidity was neutralized with small portions of NaHCO_3_ saturated solution. The biphasic solution was evaporated in vacuo to eliminate THF and the residue extracted three times with EtOAc/*brine*. The derived organic extracts were dried over anhydrous Na_2_SO_4_ and solvent evaporated in vacuo. The resulting crude product was purified over prewashed silica gel (50:1) by elution with petroleum ether/AcOEt (9:1) to afford pure product with the yield reported.

**4a**: *bis(2-(2-methoxybenzo[1,3]dioxol-5-yl)ethyl) oxalate*. Yield: 78% yellow oil; IR (cm^−1^): 3036, 3024, 2965, 2836, 1765, 1746, 1446, 1316, 1206. ^1^H-NMR (CDCl_3_) δ: 6.83 (s, 2H, CH(O)_3_); 6.79 (d, 2H, *J* = 7.9 Hz, PhH); 6.74 (d, 2H, *J* = 1.7 Hz, PhH); 6.70 (dd, 2H, *J* = 7.9, 1.7 Hz, PhH); 4.43 (t, 4H, *J* = 7.0 Hz, CH_2_O); 3.40 (s, 6H, OCH_3_); 2.97 (t, 4H, *J* = 7.0 Hz CH_2_Ph). ^13^C-NMR (CDCl_3_) δ: 157.52; 146.29; 145.02; 130.53; 122.05; 119.24; 108.93; 108.08; 67.45; 50.01; 34.48. HRMS: found, 446.1213, C_22_H_22_O_10_ requires 446.1213.

**4b**: *bis(2-(2-methoxybenzo[1,3]dioxol-5-yl)ethyl) malonate*. Yield: 55% yellow oil; IR (cm^−1^): 3029; 2939; 2836; 1732; 1512; 1448; 1223; 1075; 1029. ^1^H-NMR (CDCl_3_) δ: 6.83 (s, 2H, CH(O)_3_); 6.80 (d, 2H, *J* = 7.9 Hz, PhH); 6.75 (d, 2H, *J* = 1.7 Hz, PhH); 6.70 (dd, 2H, *J* = 7.9, 1.7 Hz, PhH); 4.24 (t, 4H, *J* = 7.0 Hz, CH_2_O); 3.39 (m, 8H, OCH_3_ e CH_2_C=O); 2.88 (m, 4H, CH_2_Ph). ^13^C-NMR (CDCl_3_) δ: 172.18; 146.12; 144.71; 131.50; 121.90; 119.12; 108.87; 107.93; 65.36; 50.00; 34.77; 29.01. HRMS: found, 460.1371, C_23_H_24_O_10_ requires 460.1369.

**4c**: *bis(2-(2-methoxybenzo[1,3]dioxol-5-yl)ethyl) succinate*. Yield: 80%, yellow oil; IR (cm^−1^): 3034; 2959; 2928; 2856 1733; 1520; 1439; 1219; 1176; 1093; 1036. ^1^H-NMR (CDCl_3_) δ: 6.83 (s, 2H, CH(O)_3_); 6.80 (d, 2H, *J* = 7.9 Hz, PhH); 6.76 (d, 2H, *J* = 1.7 Hz, PhH); 6.70 (dd, 2H, *J* = 7.9, 1.7 Hz, PhH); 4.24 (t, 4H, *J* = 7.0 Hz, CH_2_O); 3.40 (s, 6H, OCH_3_); 2.86 (t, 4H, *J* = 7.0 Hz CH_2_Ph); 2.60 (s, 4H, CH_2_C=O). ^13^C-NMR: (CDCl_3_) δ: 172.34; 146.27; 144.88; 131.66; 122.06; 119.28; 109.04; 108.09; 65.46; 50.10; 34.93; 29.18. HRMS: found, 474.1525, C_24_H_26_O_10_ requires 474.1526.

**4d**: *bis(2-(2-methoxybenzo[1,3]dioxol-5-yl)ethyl) glutarate*. Yield: 81%, yellow oil; IR (cm^−1^): 3031; 3024; 3012; 2915, 1755; 1498; 1442; 1253; 1223; 1142; 1094. ^1^H-NMR (CDCl_3_) δ: 6.83 (s, 2H, CH(O)_3_); 6.79 (d, 2H, *J* = 7.9 Hz, PhH); 6.75 (d, 2H, *J* = 1.4 Hz, PhH); 6.70 (dd, 2H, *J* = 7.9, 1.4 Hz, PhH); 4.23 (t, 4H, *J* = 7.0 Hz, CH_2_O); 3.40 (s, 6H, OCH_3_); 2.86 (t, 4H, *J* = 7.0 Hz CH_2_Ph); 2.33 (t, 4H, *J* = 7.0 Hz, CH_2_C=O); 1.90 (q, 2H, *J* = 7.0 Hz, CH_2_CH_2_C=O). ^13^C-NMR: (CDCl_3_) δ: 172.80; 146.32; 144.75; 131.75; 122.03; 119.33; 108.99; 108.07; 65.16; 50.13; 35.02; 33.38; 20.20. HRMS: found, 488.1682, C_25_H_28_O_10_ requires 488.1682.

**4e**: *bis(2-(2-methoxybenzo[1,3]dioxol-5-yl)ethyl) adipate*. Yield: 77%, yellow oil; IR (cm^−1^): 3032, 2973, 2945, 1730, 1499, 1440, 1248, 1194, 1036. ^1^H-NMR (CDCl_3_) δ: 6.82 (s, 2H, CH(O)_3_); 6.80 (d, 2H, *J* = 7.9 Hz, PhH); 6.76 (d, 2H, *J* = 1.5 Hz, PhH); 6.70 (dd, 2H, *J* = 7.9, 1.5 Hz, PhH); 4.23 (t, 4H, *J* = 7.0 Hz, CH_2_OC=O); 3.40 (s, 6H, OCH_3_); 2.86 (t, 4H, *J* = 7.0 Hz CH_2_Ph); 2.30 (m, 4H, m, CH_2_C=O); 1.62 (m, 4H, m, CH_2_CH_2_C=O). ^13^C-NMR: (CDCl_3_) δ: 173.29; 146.30; 144.89; 131.79; 122.04; 119.33; 109.01; 108.07; 65.10; 50.15; 35.03; 34.03; 24.50. HRMS: found, 502.1838, C_26_H_30_O_10_ requires 502.1839.

**4f**: *bis(2-(2-methoxybenzo[1,3]dioxol-5-yl)ethyl) suberoate*. Yield: 78%, yellow oil; IR (cm^−1^): 3031, 3024, 3012, 2930, 1730, 1439, 1420, 1253, 1223, 1160, 1030. ^1^H-NMR (CDCl3) δ: 6.83 (s, 2H, CH(O)_3_); 6.79 (d, 2H, *J* = 7.9 Hz, PhH); 6.76 (s, 2H, PhH); 6.71 (d, 2H, *J* = 7.9 Hz, PhH); 4.23 (t, 4H, *J* = 6.9 Hz, CH_2_OC=O); 3.40 (s, 6H, OCH_3_); 2.86 (t, 4H, *J* = 6.9 Hz CH_2_Ph); 2.28 (t, 4H, *J* = 7.5, CH_2_C=O); 1.58 (m, 4H, CH_2_CH_2_C=O); 1.29 (m, 4H, CH_2_CH_2_CH_2_C=O). ^13^C-NMR (CDCl_3_) δ: 173.75; 146.26; 144.85; 131.83; 122.06; 119.29; 109.04; 106.06; 65.02; 50.14; 35.03; 34.03; 28.87; 24.85. HRMS: found, 530.2151, C_28_H_34_O_10_ requires 530.2152.

**4g**: *bis(2-(2-methoxybenzo[1,3]dioxol-5-yl)ethyl) trans-1,4-cyclohexandicarboxylate*. Yield: 80%, yellow oil; IR (cm^−1^): 3031, 3024, 3014, 2930, 1730, 1442, 1420, 1250, 1223, 1160, 1030. ^1^H-NMR (CDCl_3_) δ: 6.83 (s, 2H, CH(O)_3_); 6.80 (d, 2H, *J* = 7.9 Hz, PhH); 6.75 (s, 2H, PhH); 6.69 (d, 2H, *J* = 7.9 Hz, PhH); 4.23 (t, 4H, *J* = 6.8 Hz, CH_2_OC=O); 3.40 (s, 6H, OCH_3_); 2.86 (t, 4H, *J* = 6.8 Hz, CH_2_Ph); 2.25 (m, 2H, CHC=O); 1.99 (m, 4 H, CHeqCHC=O); 1.41 (m, 4H, CHaxCHC=O). ^13^C-NMR (CDCl_3_) δ: 175.45; 146.28; 144.88; 131.77; 122.06; 119.31; 109.02; 106.06; 65.04; 50.13; 42.61 (CH_2_C=O); 35.03 (CH_2_Ph); 28.11 (CH_2_CH_2_C=O). HRMS: found, 528.1994, C_28_H_32_O_10_ requires 528.1995.

**4h**: *bis(2-(2-methoxybenzo[1,3]dioxol-5-yl)ethyl) fumarate*. Yield: 75%, yellow oil; IR (cm^−1^): 3031, 3024, 3012, 2930, 1715, 1498, 1420, 1315, 1260, 1253, 1223, 980, 870, 750. ^1^H-NMR (CDCl_3_) δ: 6.84 (s, 2H, CH(O)_3_); 6.81 (s, 2H, HC=C), 6.80 (d, 2H, *J* = 7.8 Hz, PhH); 6.77 (s, 2H, PhH); 6.72 (d, 2H, *J* = 7.9, PhH); 4.36 (t, 4H, *J* = 8.8 Hz, CH_2_OC=O); 3.40 (s, 6H, OCH_3_); 2.92 (t, 4H, *J* = 6.7 Hz, CH_2_Ph).^13^C-NMR (CDCl_3_) δ: 164.93; 146.35; 144.99; 133.73; 131.32; 122.07; 119.33; 106.96; 106.15; 66.06; 50.14; 34.85. HRMS: found, 472.1369, C_24_H_24_O_10_ requires 472.1369.

**4i**: *bis(2-(2-methoxybenzo[1,3]dioxol-5-yl)ethyl) phthalate*. Yield: 76%, yellow oil; IR (cm^−1^): 3035, 3010, 2965, 2870, 1736, 1680, 1612, 1496, 1295, 1288, 1250. ^1^H-NMR (CDCl_3_) δ: 7.66 (dd, 2H, *J* = 3.3, 5.5 Hz, PhH); 7.52 (dd, 2H, *J* = 3.3, 5.5 Hz, PhH); 6.81 (s, 2H, CH(O)_3_); 6,79 (d, 2H, *J* = 8.0, PhH); 6.78 (s, 2H, PhH); 6.73 (d, 2H, *J* = 8.0 Hz, PhH); 4.41 (t, 4H, *J* = 7.0 Hz, CH_2_OC=O); 3.39 (s, 3H, CH_3_O); 2.95 (t, 4H, *J* = 7.0 Hz, CH_2_Ph). ^13^C-NMR (CDCl_3_) δ: 167.54; 146.32; 144.93; 136.14; 131.61; 131.21; 129.01; 122.13; 119.31; 109.06; 106.11; 66.35; 50.12; 34.85. HRMS: found, 522.1525, C_28_H_26_O_10_ requires 522.1526.

**4j**: *bis(2-(2-methoxybenzo[1,3]dioxol-5-yl)ethyl) isophthalate*. Yield: 75%, yellow oil; IR (cm^−1^): 3034, 3011, 2962, 1735, 1680, 1610, 1498, 1290, 1288, 1249. ^1^H-NMR (CDCl_3_) δ: 8.65 (s, 1H, PhH), 8.19 (d, 2H, *J* = 7.7 Hz, PhH), 7.52 (t, 1H, *J* = 7.7 Hz, PhH), 6.83 (s, 4H, CH(O)_3_ e PhH); 6,82 (d, 2H, *J* = 7.4 Hz, PhH); 6.78 (d, 2H, *J* = 7.4 Hz, PhH); 4.51 (t, 4H, *J* = 7.0 Hz, CH_2_OC=O); 3.40 (s, 3H, CH_3_O); 3.03 (t, 4H, *J* = 7.0 Hz, CH_2_Ph). ^13^C-NMR (CDCl_3_) δ: 165.71; 146.36; 144.97; 133.91; 131.60; 130.80; 130.82; 128.76; 122.14; 119.32; 109.00; 108.14; 66.06; 50.08; 35.10. HRMS: found, 522.1526, C_28_H_26_O_10_ requires 522.1526.

### 3.4. Deprotection of ***4a–4j*** to Products ***2a–2j***

In addition, 0.3 mmoles of protected ester (**4a–4j**) were dissolved in 9 mL of anhydrous THF. 250 mg of Amberlyst 15 acidic resin and a small quantity of phosphate buffer, made previously by equimolar K_2_HPO_4_/KH_2_PO_4_, were added, and soon after 20 equivalents of anhydrous methanol.

The reaction was controlled by HPLC analysis and when all the substrate is deprotected (generally 2 h) the pH of the reaction mixture was monitored and eventually neutralize with NaHCO_3_ aqueous solution. The solution was filtered to remove the resin, and THF evaporated in vacuo. The raw material was diluted with EtOAc/*brine* and the product was extracted. Organic phases were dried over anhydrous Na_2_SO_4_ and solvent evaporated in vacuo. When necessary (compound **2h**), the resulting crude product was purified over pre-washed silica gel (50:1) by elution with petroleum ether/AcOEt (7:3) to afford pure product with the yield reported.

**2a**: *bis(3,4-dihydroxyphenethyl) oxalate*. Yield: 89%, yellow oil; IR (cm^−1^): 3553, 2959, 2929, 2871, 2857, 1765, 1732, 1525, 1410, 1374, 1261. ^1^H-NMR (CDCl_3_) δ: 6.66 (d, 2H, *J* = 8.0Hz, PhH); 6.64 (d, 2H, *J* = 2.0 Hz, PhH); 6.52 (dd, 2H *J* = 8.0, 2.0 Hz, PhH); 4.36 (t, 4H, *J* = 7.0 Hz, CH_2_O); 2.84 (t, 4H, *J* = 7.0 Hz, CH_2_Ph). ^13^C-NMR (CDCl_3_) δ: 159.19; 146.37; 145.16; 130.03; 121.38; 117.11; 116.50; 64.64; 35.12. HRMS: found, 362.1002, C_18_H_18_O_8_ requires 362.1002.

**2b**: *bis(3,4-dihydroxyphenethyl) malonate*. The reaction rapidly produced a dark solution with a black pitchy precipitate. After work-up, the extracted solution presented no evidence of reagent and product. Different procedures did not solve the degradation problems.

**2c**: *bis(3,4-dihydroxyphenethyl) succinate*. Yield: 86%, yellow oil; IR (cm^−1^): 3553, 2959, 2929, 2871, 2857, 1732, 1525, 1450; 1374, 1261, 1170. ^1^H-NMR (CDCl_3_) δ: 6.71 (d, 2H, *J* = 10.0 Hz, PhH); 6.63 (d, 2H, *J* = 2.0 Hz, PhH); 6.50 (dd, 2H, *J* = 10.0, 2.0 Hz, PhH); 4.15 (t, 4H, *J* = 7.0 Hz, CH_2_O); 2.71 (t, 4H, *J* = 7.0 Hz, CH_2_Ph); 2,53 (s, 4H, CH_2_CO). ^13^C-NMR: (CD_3_OD) δ: 159.13; 146.32; 145.10; 131.76; 129.97; 121.24; 117.02; 68.68; 39.65; 35.08. HRMS: found 390.1314, C_20_H_22_O_8_ requires 390.1315.

**2d**: *bis(3,4-dihydroxyphenethyl) glutarate*. Yield: 88%, yellow oil; IR (cm^−1^): 3557, 2949, 2873, 2861, 1757, 1527, 1413,1375, 1255, 1195. ^1^H-NMR: (CDCl_3_) δ: 6.80 (d, 2H, *J* = 8.0 Hz, PhH); 6.73 (d, 2H, *J* = 2.0 Hz, PhH); 6.63 (dd, 2H, *J* = 8.0, 2.0 Hz, PhH); 4.28 (t, 4H, *J* = 7.0 Hz CH_2_O); 2.82 (t, 4H, *J* = 7.0 Hz CH_2_Ph); 2.30 (s, 4H, *J* = 7.0 Hz, CH_2_C=O); 1.90 (q, 2H, *J* = 7.0 Hz, CH_2_CH_2_C=O). ^13^C-NMR: (CD_3_OD) δ: 173.88; 145.30; 143.98; 129.82; 120.34; 116.09; 115.46; 65.64; 34.55;33.15; 20.36. HRMS: found 404.1470, C_21_H_24_O_8_ requires 404.1471.

**2e**: *bis(3,4-dihydroxyphenethyl) adipate*. Yield: 92%, yellow oil; IR (cm^−1^): 3555, 2932, 2863, 1729, 1535, 1398, 1369, 1245. ^1^H-NMR: (CD_3_OD) δ: 6.66 (d, 2H, *J* = 8.1 Hz, PhH); 6.64 (d, 2H, *J* = 2.0 Hz, PhH); 6.51 (dd, 2H, *J* = 8.1,2.0 Hz, PhH); 4.18 (t, 4H, *J* = 7.0 Hz CH_2_O); 2.74 (t, 4H, *J* = 7.0 Hz CH_2_Ph); 2.26 (m, 4H, m, CH_2_C=O); 1.56 (m, 4H, m CH_2_CH_2_C=O). ^13^C-NMR: (CDCl_3_) δ: 173.29; 146.30; 144.89; 131.79; 122.04; 119.33; 109.01; 108.07; 65.10; 50.15; 35.03; 34.03; 24.50. HRMS: found, 418.1628, C_22_H_26_O_8_ requires 418.1628.

**2f**: *bis(3,4-dihydroxyphenethyl) octanedioate*. Yield: 88%, yellow oil; IR (cm^−1^): 3556, 2930, 2861; 1729, 1535, 1399, 1369, 1246. ^1^H-NMR (CDCl_3_) δ: 6.64 (d, 2H, *J* = 8.0 Hz, PhH); 6.62 (s, 2H, PhH); 6.49 (d, 2H, *J* = 8.0 Hz, PhH); 4.16 (t, 4H, *J* = 6.9 Hz, CH_2_O); 2.72 (t, 4H, *J* = 6.9 Hz, Ch_2_Ph); 2.24 (m, 4H, C_2_H); 1.50 (m, 4H, C_3_H); 1.20 (m, 4H, C_4_H). ^13^C-NMR (CDCl_3_) δ: 175.60; 146.21; 144.89; 130.75; 121.18; 116.99; 116.33; 66.39; 35.45; 35.03; 29.70; 25.82. HRMS: found, 446.1941, C_24_H_30_O_8_ requires 446.1941.

**2g**: *bis(3,4-dihydroxyphenethyl) trans-1,4-ciclohexandicarboxylate*. Yield: 90%, yellow oil; IR (cm^−1^) 3556, 3032, 3020, 2932, 2863; 1729, 1535, 1398; 1370, 1245, 1161, 1033. ^1^H-NMR (CDCl_3_) δ: 6.68 (d, 2H, *J* = 8.0 Hz, PhH); 6.62 (d, 2H, *J* = 1.8 Hz, PhH); 6.53 (dd, 2H *J* = 8.0, 1.8 Hz, PhH); 4.16 (t, 4H, *J* = 6.8 Hz, CH_2_OC=O); 2.72 (t, 4H, *J* = 6.8 Hz, CH_2_Ph); 2.21 (m, 2H, C_2_H); 1.93 (m, 4H, C_3_H); 1.34 (m, 4H, C_3_H).^13^C-NMR (CDCl_3_) δ: 177.19; 146.21; 144.90; 130.74; 121.21; 117.04; 116.33; 66.43; 43.78; 35.46; 29.12. HRMS: found, 444.1784, C_24_H_28_O_8_ requires 444.1784.

**2h**: *bis(3,4-dihydroxyphenethyl) fumarate*. Yield: 65%, yellow oil; IR (cm^−1^): 3555, 3035, 3016, 2932, 2865, 2846; 1715, 1587, 1443; 1314, 1252, 1150. ^1^H-NMR (CDCl_3_) δ: 6.73 (s, 2H, HC=C); 6.65 (d, 2H, *J* = 8.0 Hz, PhH); 6.63 (d, 2H, *J* = 1,7 Hz, PhH); 6.51 (dd, 2H, *J* = 8.0, 1,7 Hz, PhH); 4.28 (t, 4H, *J* = 6.9 Hz, CH_2_OC=O); 2.79 (t, 4H, *J* = 6,9 Hz, CH_2_Ph).^13^C-NMR (CDCl_3_) δ: 166.25; 146.29; 145.01; 134.54; 130.43; 121.23; 116.49; 116.41; 67.42; 35.30. HRMS: found, 388.1158, C_20_H_20_O_8_ requires 388.1158

**2i**: *bis(3,4-dihydroxyphenethyl) phthalate*. Yield: 95%, pale yellow oil; IR (cm^−1^): 3550; 3035, 3016, 2930, 2865, 2847; 1730, 1587, 1440; 1312, 1250. ^1^H-NMR (CDCl_3_) δ: 7.63 (dd, 1H, *J* = 3.3, 5.6 Hz, PhH), 7.56 (dd, 1H, *J* = 3.3, 5.6 Hz, PhH), 6.67 (d, 2H, *J* = 8.0 Hz, PhH); 6.66 (s, 2H, PhH); 6.54 (dd, 2H, *J* = 1.7, 8.0 Hz, PhH); 4.32 (t, 4H, *J* = 6.7 Hz, CH_2_O); 2.82 (t, 4H, *J* = 6.7 Hz, CH_2_Ph). ^13^C-NMR (CDCl_3_) δ: 169.71; 146.26; 144.97; 133.47; 132.35; 130.64; 129.91; 121.31; 117.08; 116.43; 67.83; 35.29. HRMS: found, 438.1315, C_24_H_22_O_8_ requires 438.1315.

**2j**: *bis(3,4-dihydroxyphenethyl) isophthalate*. Yield: 99%, pale yellow oil. IR (cm^−1^): 3548, 3034, 3014, 2932, 2865, 2847; 1726, 1587, 1440; 1313, 1249. ^1^H-NMR(CDCl_3_) δ: 8.69 (s, 1H, PhH), 8.17 (d, 2H, *J* = 6.6 Hz, PhH), 7.50 (t, 1H, *J* = 6.6 Hz, PhH), 6.88 (s, 2H, PhH); 6.80 (d, 2H, *J* = 8.0 Hz, PhH); 6.68 (d, 2H *J* = 8.0 Hz, PhH); 4.48 (t, 4H, *J* = 6.6 Hz, CH_2_O); 2.95 (t, 4H, *J* = 6.6 Hz, CH_2_Ph). ^13^C-NMR (CDCl_3_) δ: 167.95; 145.30; 143.98; 134.07; 131.29; 130.68; 129.03; 121.93; 121.58; 116.77; 115.56; 66.47; 34.74. HRMS: found, 438.1315, C_24_H_22_O_8_ requires 438.1315.

### 3.5. Partition Coefficient Values

Log P values of all compounds were calculated by Chem BIO Office 2010© [29] (Table 2). As compounds have never been synthesized, no experimental data are available by literature.

### 3.6. ABTS Assay

The antioxidant capacities of compounds **2a–2j** were measured according to the method of Pellegrini et al. but at room temperature as described elsewhere [13]. In brief, the analyses were performed in ethanol (0.2% of water), measuring the absorbance at 734 nm using a PerkinElmer Lambda 14P spectrophotometer. ABTS^•+^ solution in H_2_O was diluted with EtOH until the absorbance of 0.70 ± 0.20. Then, 10 μL of sample was added to 0.99 mL of ABTS solution. Trolox, as reference standard, and esters **2a–2j** were analyzed at four concentrations from 1 to 10 μM. The plateau of radical quenching was obtained after 3 min. Each concentration was recorded in quadruplicate. Solvent mixture was used as blank. Collected data showed SD always below 3%. The radical-scavenging capacity of each samples was expressed as % reduction of ABTS^•+^ absorbance according to the following equation: % reduction = [(A_control_ − A_sample_)/A_control_] × 100. The percentages of ABTS inhibition vs. the amount of antioxidant concentration were used to draw the dose–response curves and linear regression was calculated using Prism 4.1 software (Graph Pad, San Diego, CA, USA). Trolox equivalent antioxidant capacity (TEAC, mmol/L) was used to report the antioxidant properties of compounds. Results are expressed as means ± standard deviation and are reported in Figure 1. Statistical analyses were performed by applying Student’s test. The level of significance was *p* < 0.05 for all data.

### 3.7. DCF Assay

The applied method was a standard assay based on the intracellular fluorescent probe DCF [30,32]. The antioxidant activities of each compound were analyzed in L6 and THP-1 cell culture, using an intracellular DCF fluorescent probe on cells exposed to CH oxidative stress. Fluorescence was measured over a 10 min period, and the ability of the compounds to eliminate the increased ROS production was monitored. L6 myoblasts and THP-1 monocytes grown and analyses were carried out as described elsewhere [14]. Intracellular fluorescence was measured at 37 °C using a PerkinElmer VICTOR 3V spectrometer. Excitation and emission wavelengths were set at λ = 498 and 530 nm, respectively. CH in DMSO was used as radical generator (final concentration= 200 μM); DMSO at the used concentrations did not affect the fluorescence signal. Cells were incubated with the compounds at final concentration of 10 μM for 10 min at 37 °C before the addition of CH. The decreases in the intracellular DCF fluorescence, reported as ΔF/10 min, determined the antioxidant activity and were calculated relative to the fluorescence change induced by 200 μM CH alone (100%). Data are reported as the mean ± standard deviation of at least *n* = 4 different experiments and reported as numerical data in Table SM1, and Figure 2 and Figure 3.

One-way ANOVA test (nonparametric) and Bonferroni post-test were used as statistical analyses, comparing all pairs of columns.

### 3.8. MTT Assay

The method of Hansen et al. [33] was used with some modifications. The experiment was carried out in 96-well plate using L6-cells at a confluent state. The cells were scraped from the surface of the flask and resuspended in 5mL of DMEM supplemented with 10% fetal bovine serum, 100 µg/mL streptomycin, and 100 U/mL penicillin. The wells were seeded with 5000 cells in a final volume of 200mL and were incubated for 24 h (atmosphere of 5% CO_2_ at 37 °C). Stock solutions of **2a–2j** were prepared in ethanol and subsequently diluted in DMEM supplemented with 10% fetal bovine serum, 100 µg/mL streptomycin and 100 U/mL penicillin to final concentrations of 160 µM, 80 µM, 40 µM, 20 µM, 10 µM, and 5 µM. After 24 h incubation, DMEM was discarded from the wells and 100 µL of the test solutions were added. The cells were incubated for 24 h (atmosphere of 5% CO_2_ at 37 °C). Each compound was tested in triplicate and in control wells was added medium without antioxidant. After incubation, the medium was discarded from the wells, and the cells were washed with 1 mL/well of PBS + glucose at 37 °C. The controlled and treated cells were incubated with 100 µL of MTT solution at final concentration of 1 mg/mL for 3 h (atmosphere of 5% CO_2_ at 37 °C). Lysing buffer was added and after 2 days MTT formazan was measured with a spectrophotometer at 560 nm.

### 3.9. Proliferation Curves

Proliferation curves were performed as previously reported [14]. In brief, either L6 or THP1 cells were seeded in the appropriate medium and after 24 h, the cells were exposed to the hydroxytyrosyl esters **2g–2j** (10 μM final concentration) with or without CH (40 μM in L6 and 200 μM in THP-1 cell culture). Neubauer chamber was always used to count cells every 24 h, up to confluence. In case of L6 cells, a mild trypsinization was carried out before cells counting. The results are given as the mean ± standard deviation of two different experiments.

### 3.10. Statistical Analysis

GraphPad Prism 4.1 statistics program was used to perform data analyses. One-way ANOVA and Bonferroni post-test were used in all cell culture experiments, while Student’s *t*-test was preferred in ABTS experiments. Differences were considered significant at *p* < 0.05.

## 4. Conclusions

Nine new dihydroxytyrosyl esters **2a, 2c–2j** were obtained in high yield using the esterification of orthoformate protected HTyr **3** with the corresponding acyl chlorides and the following deprotection under mild conditions. These new antioxidants were tested in vitro by ABTS and by DFC assay on two cell lines. The experimental data confirmed the high radical scavenging ability of all synthesized compounds. However, the results evidenced a dichotomy between in vitro and on cell culture experiments. According to the ABTS assay, the best performances were obtained by less lipophilic dihydroxytyrosyl oxalate **2a** and succinate **2c**, probably because of conformational effects, that favor antioxidants with lower distances between catechol moieties. Conversely, in cell cultures, dihydroxytyrosyl esters of 1,4-trans-ciclohexandicarboxylate **2g**, fumarate **2h**, phthalate **2i**, and isophthalate **2j** produced the higher ROS protection. The performance of the dihydroxytyrosyl antioxidants **2g–2j** in the cellular environment might be explained by a balance between the lipophilicity that favors the membrane penetration, and the structure rigidity that permits the release into the lumen where the antioxidant effect is detected by the fluorescent probe. To evaluate possible uses as preservatives, MTT assay and proliferation curves were also evaluated, confirming very low toxicity of all compounds tested. The complete comprehension of the cell absorption phenomenon will be examined in depth in the future research.

## Figures and Tables

**Table 1:** List of designed dihydroxytyrosyl ester **2a–2j** with their dicarboxylic moieties.

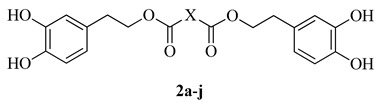		
Compound	Dicarboxylic Moiety	-COXCO-Residue
**2a**	Oxalyl	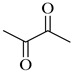
**2b**	Malonyl	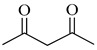
**2c**	Succinyl	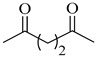
**2d**	Glutaryl	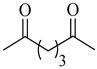
**2e**	Adipoyl	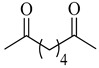
**2f**	Suberoyl	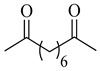
**2g**	1,4-trans-Cyclohexandicarbossyl	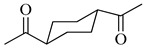
**2h**	Fumaryl	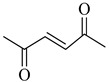
**2i**	Phthalyl	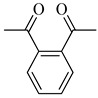
**2j**	Isophthalyl	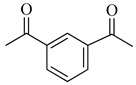

**Table 2:** Molecular weight, Log P and TEAC values of HTyr **1** and dihydroxytyrosyl esters **2a, 2c–2j**. LogP were calculated by Chem BIO Office 2010© [29]. Log P and TEAC data are reported with their standard deviation.

Compound	MW	LogP©	± SD	TEAC	± SD
**1**	154	0.96	± 0.5	0.79	± 0.03
**2a**	362	2.47	± 0.5	2.0	± 0.2
**2c**	390	2.35	± 0.5	1.7	± 0.2
**2d**	404	2.77	± 0.5	1.6	± 0.1
**2e**	418	3.19	± 0.5	1.4	± 0.2
**2f**	446	4.02	± 0.5	1.12	± 0.08
**2g**	444	3.82	± 0.5	1.3	± 0.1
**2h**	388	2.63	± 0.5	1.3	± 0.1
**2i**	438	4.14	± 0.5	1.7	± 0.1
**2j**	438	4.14	± 0.5	1.5	± 0.1

## Supplementary Materials

The following are available online. **Figure SM1**. TEAC vs. LogP. Correlation between LogP and TEAC values for saturated derivatives **2a, 2c–2f**. **Table SM1**. DCF assay of **1** and **2a, 2c–2j**. **Figure SM2**. Effect of **2f** (10 μM) on the proliferation of L6 and THP-1 cells with or without CH. **Figure SM2**. Effect of **2h** (10 μM) on the proliferation of L6 and THP-1 cells in the absence and in the presence of CH. **Figure SM3**. Effect of **2i** (10 μM) on the proliferation of L6 and THP-1 cells in the absence and in the presence of CH. **Figure SM2**. Effect of **2j** (10 μM) on the proliferation of L6 and THP-1 cells in the absence and in the presence of CH.
