# Supplementary material for: New Dihydroxytyrosyl Esters from Dicarboxylic Acids: Synthesis and Evaluation of the Antioxidant Activity In Vitro (ABTS) and in Cell-Cultures (DCF Assay)
Source: Molecules. 2020 Jul 9;25(14):3135. doi: 10.3390/molecules25143135 (PMC7397168; doi:10.3390/molecules25143135)
Supplement: Supplementary file 1 [file molecules-25-03135-s001.pdf]

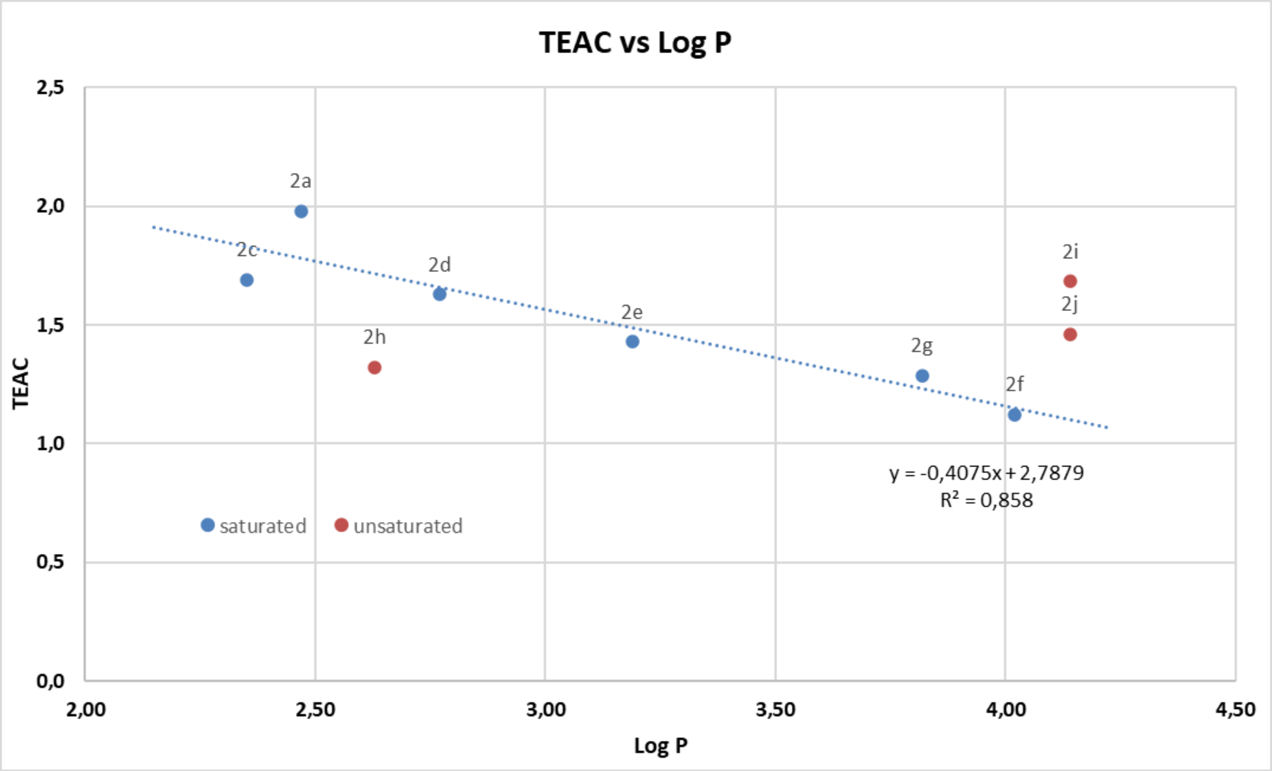

Figure SM1. TEAC vs Log P. Data evidence the inverse linear correlation between antioxidant capacities and lipophilicity in hydroxytyrosyl esters **2a,c-f.**

Table SM1. Determination of intracellular ROS in L6 and THP1 cell lines after stimulation of DCF fluorescence with 40 (L6) or 200 μM (THP-1) cumene hydroperoxide alone (CH) and in presence of dihydroxytyrosyl esters **2a,c-j** at 10 and 1μM concentrations. Data are reported as mean values ± SD of five experiments carried out in triplicate. Statistical analysis performed with one-way ANOVA and Bonferroni post-test.

	L6 cell culture		THP1 cell culture	
**conc**	**1μM**	**10μM**	**1μM**	**10μM**
**compound**	**% ROS**	**% ROS**	**% ROS**	**% ROS**
**CHP**	100 ±5	100 ±5	100 ±5	100 ±5
**1**	62 ±6	30 ±8	62 ±15	38 ±20
**2a**	58 ±17	34 ±17	56 ±13	35 ±12
**2c**	35 ±19	24 ±14	55 ±12	33 ±11
**2d**	41 ±20	20 ±12	50 ±18	25 ±10
**2e**	37 ±18	12 ±10	56 ±18	22 ±5
**2f**	2 ±6	2 ±4	28 ±4	21 ±8
**2g**	8 ±4	1 ±2	22 ±6	10 ±3
**2h**	6 ±8	2 ±7	15 ±10	5 ±6
**2i**	10 ±16	11 ±11	18 ±7	8 ±3
**2j**	34 ±15	7 ±9	15 ±4	3 ±4

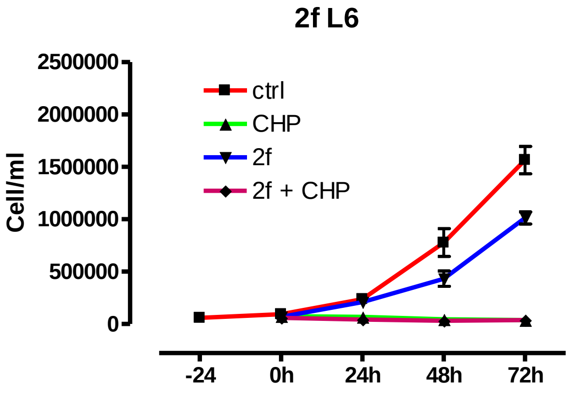

(**a**)

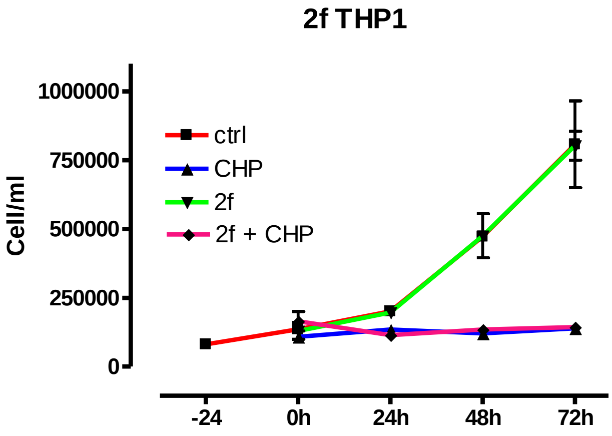

(**b**)

Figure SM2. Effect of compound **2f** (10 μM) on the proliferation of L6 (**a**) and THP-1(**b**) cells in presence or absence of cumene hydroperoxide (CH, 40 μM in L6 and 200 μM in THP-1 cells). Cell counting was done with a Neubauer chamber. Data are reported as mean values ± SD of each compound tested in duplicate. Statistical analysis performed with one-way ANOVA and Bonferroni post-test.

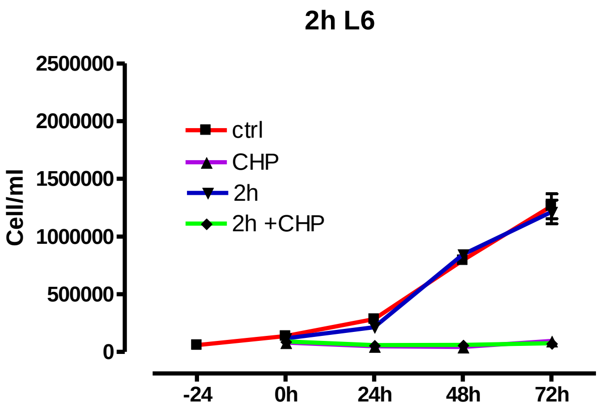

(**a**)

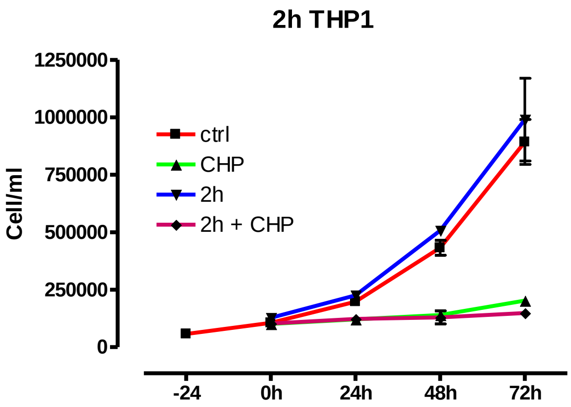

(**b**)

Figure SM3. Effect of compound **2h** (10 μM) on the proliferation of L6 (**a**) and THP-1(**b**) cells in presence or absence of cumene hydroperoxide (CH, 40 μM in L6 and 200 μM in THP-1 cells). Cell counting was done with a Neubauer chamber. Data are reported as mean values ± SD of each compound tested in duplicate. Statistical analysis performed with one-way ANOVA and Bonferroni post-test.

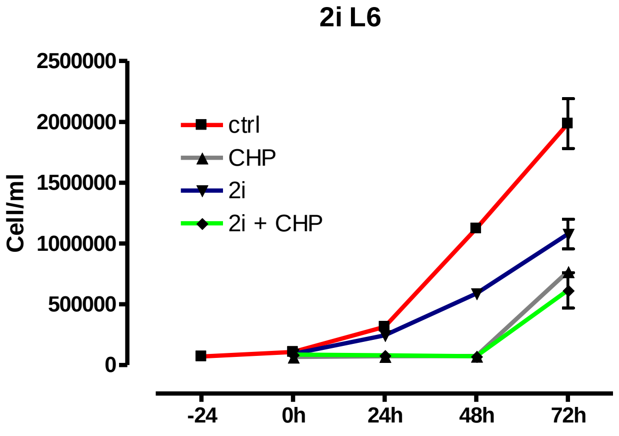

(**a**)

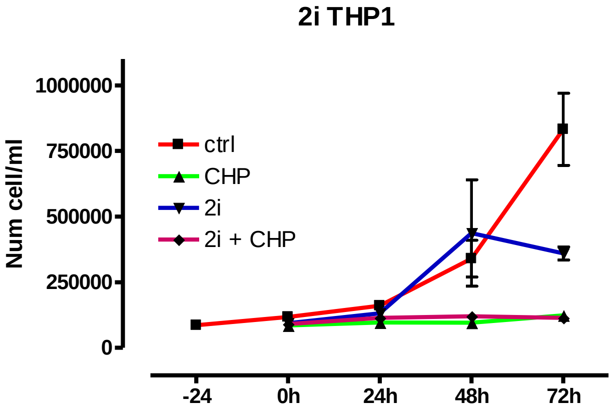

(**b**)

Figure SM4. Effect of compound **2i** (10 μM) on the proliferation of L6 (**a**) and THP-1(**b**) cells in presence or absence of cumene hydroperoxide (CH, 40 μM in L6 and 200 μM in THP-1 cells). Cell counting was done with a Neubauer chamber. Data are reported as mean values ± SD of each compound tested in duplicate. Statistical analysis performed with one-way ANOVA and Bonferroni post-test.

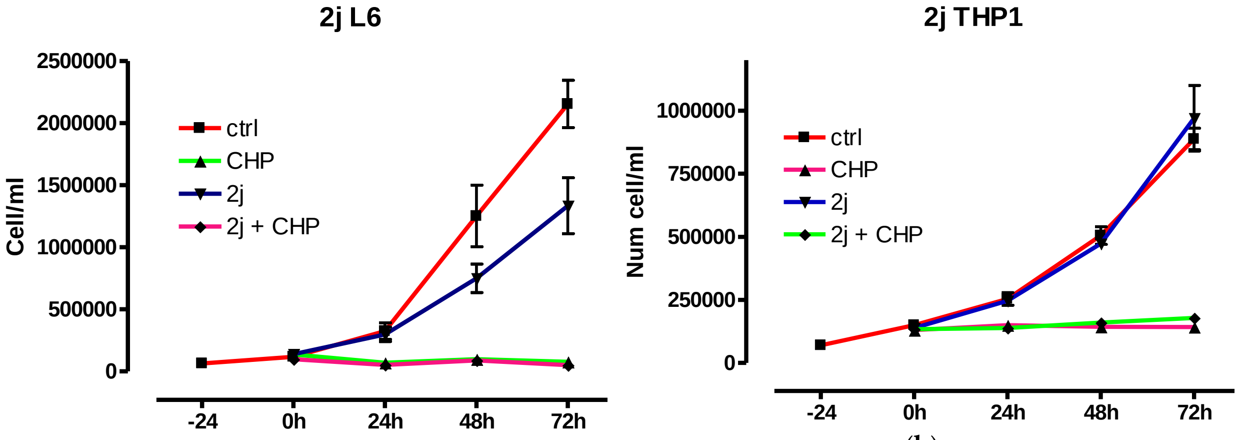

(**a**) (**b**)

Figure SM5. Effect of compound **2j** (10 μM) on the proliferation of L6 (**a**) and THP-1(**b**) cells in presence or absence of cumene hydroperoxide (CH, 40 μM in L6 and 200 μM in THP-1 cells). Cell counting was done with a Neubauer chamber. Data are reported as mean values ± SD of each compound tested in duplicate. Statistical analysis performed with one-way ANOVA and Bonferroni post-test.
